# Evaluation of the collaborative network of highly correlating skin proteins and its change following treatment with glucocorticoids

**DOI:** 10.1186/1742-4682-7-16

**Published:** 2010-05-28

**Authors:** Uwe Klinge, Nicolette Farman, Anette Fiebeler

**Affiliations:** 1 Surgical Department of the University Hospital of the RWTH Aachen, Germany; 2 Department for applied medical engineering, University Hospital of the RWTH Aachen, Germany; 3 INSERM, Collège de France, Paris, INSERM U 872, équipe 1, Centre de Recherche des Cordeliers, Université Pierre et Marie Curie, France; 4 Clinics for Nephrology and Hypertension at the Hannover Medical School, Germany

## Abstract

**Background:**

Glucocorticoids (GC) represent the core treatment modality for many inflammatory diseases. Its mode of action is difficult to grasp, not least because it includes direct modulation of many components of the extracellular matrix as well as complex anti-inflammatory effects. Protein expression profile of skin proteins is being changed with topical application of GC, however, the knowledge about singular markers in this regard is only patchy and collaboration is ill defined.

**Material/Methods:**

Scar formation was observed under different doses of GC, which were locally applied on the back skin of mice (1 to 3 weeks). After euthanasia we analyzed protein expression of collagen I and III (picrosirius) in scar tissue together with 16 additional protein markers, which are involved in wound healing, with immunhistochemistry. For assessing GC's effect on co-expression we compared our results with a model of random figures to estimate how many significant correlations should be expected by chance.

**Results:**

GC altered collagen and protein expression with distinct results in different areas of investigation. Most often we observed a reduced expression after application of low dose GC. In the scar infiltrate a multivariate analysis confirmed the significant impact of both GC concentrations. Calculation of Spearman's correlation coefficient similarly resulted in a significant impact of GC, and furthermore, offered the possibility to grasp the entire interactive profile in between all variables studied. The biological markers, which were connected by significant correlations could be arranged in a highly cross-linked network that involved most of the markers measured. A marker highly cross-linked with more than 3 significant correlations was indicated by a higher variation of all its correlations to the other variables, resulting in a standard deviation of > 0.2.

**Conclusion:**

In addition to immunohistochemical analysis of single protein markers multivariate analysis of co-expressions by use of correlation coefficients reveals the complexity of biological relationships and identifies complex biological effects of GC on skin scarring. Depiction of collaborative clusters will help to understand functional pathways. The functional importance of highly cross-linked proteins will have to be proven in subsequent studies.

## Introduction

For more than 50 years glucocorticoids (GC) have been studied as important adrenal hormones, which affect many physiological responses. Because of marked anti-inflammatory effects their local application remains the therapy of choice for many diseases of the skin. However, recent findings about interaction of GC with the mineralocorticoid receptor, which stimulates pro-inflammatory effects, suggest more contradicting actions [1]. For optimizing concentration and mode of application it is essential to specify the characteristic effects of GC on skin, which might be used as guide for further developments.

A characterization of the effects of GC is straightforward in simple systems, investigating e.g. GC as sole agent on a clear readout. In such a system, regardless, whether on gene, protein or clinical level, any singular effect usually manifests after some time depending on the concentration of the agent, at best in an s-shaped configuration that strongly confirms causal relationship [2].

But as many physiological regulators, GC is integrated in several basic signaling cascades. Until now, a GC-specific marker/readout, which allows a clear qualitative assessment of its effect, has not been identified. Instead, GC show a broad field of interactions with hundreds of genes and proteins [3]. Although we have to assume that most of the interferences are not yet known, and correspondingly cannot be controlled in any experimental setting, measurement of any upstream or downstream marker has to consider the superposition of many collaborative effects, which modify its concentration by positive and negative feedback. With better knowledge of these interactions, a specific intervention to modulate activity without unwanted side effects may be developed.

To approach the issue of GC-regulated networks *in vivo *in skin, we tested whether topical application of GC results in circumscriptive and specific effects. We analyzed protein expression of all together 18 different proteins in tissue samples of mice, which had been treated for up to 3 weeks with ethanol (control), low-dose or high-dose concentration of GC, respectively. In skin scar, we investigated whether some single markers specifically reflected the change of GC-concentration. Furthermore, based on Spearman's correlation coefficients, we looked at significant co-expression, which subsequently were depicted in a collaborative network. An *in silico *simulation with random numbers representing a data set without any collaborative effect served as control and defined how many relations should be expected just by chance. Finally, we tried to identify specific conditions that indicate highly cross-linked structures.

## Materials/methods

### Animals

Female mice (B6D2, age 3-6 month) were kept according to the international guidelines for animal experiments. The local authorities approved the experiment. At least five mice were treated and investigated in each treatment group for every time point. Hair was removed and a 1 cm incision made, followed by immediate closure of it with a suture. The skin on the back was treated either with (1) 50% ethanol as control, (2) low dose - 0.1 micromolar - clobetasol (Ld-GC) in 50% ethanol, or (3) high dose - 0.1 millimolar - clobetasol(Hd-GC), in 50% ethanol (clobetasol proprionat was purchased from Sigma; it is a widely and topically used glucocorticoid in dermatology, which is a very potent agonist at the glucocorticoid receptor with weak agonistic effects at the mineralocorticoid receptor; see also effects of the comparable beclomethasol [4]). After euthanasia skin/scar samples were harvested after 1 and 2 weeks in the group with ethanol or with Hd-GC. The interval was prolonged for the Ld-GC group to 1 and 3 weeks (study protocol is shown schematically in Additional file 1, **Figure S1**.

### Histochemical analysis

For collagens we performed picrosirius staining. Collagens were measured by cross-polarization microscopy and analyzed in the scar tissue. 5 μm sections were stained for 1 h in picrosirius solution (0.1% solution of Sirius Red F3BA in saturated aqueous picric acid, pH 2) [5]. For each sample, ten regions (400×, area 100 μm × 100 μm) were captured using a digital camera (Olympus C-3030, Hamburg, Germany). Using a digital image analyzing software (Image-Pro Plus^® ^4.5, Media Cybernetics, Silver Spring, MD, USA), the number of red pixels, representing mature and cross-linked collagen type I, and of green pixels, representing immature collagen type III of early phase of wound healing, were counted.

The other markers were assessed with immunohistochemistry. Depending on marker and area investigated, some staining pattern reminded on individual status of cells, rather than unified formation of a functional unit. We defined areas of interest, which could clearly be identified in every sample and by every investigator (Additional file 2, **Figure S2**):

- stratum basilare as basal cells of the epidermis,

- stratum spinosum as row of cells close to the stratum basilare,

- the seborrhoic glands,

- cells surrounding the hair, named as hairholder,

- cell infiltrate in the dermis below the scar,

- and, eventually the thickened layer of the epidermal regeneration as top of the scar.

Two independent, blinded observers did a semi-quantitative scoring from 0 to 3: 0 = less than 5% of cells (negative, rarely), 1 = 5 to 30% (occasional, some), 2 = 30-80% (usual, many), 3 = > 80% of cells (almost all).

Histochemical procedure was done according to manufacturers protocol with the following antibodies: Axl (polyclonal, goat) - Santa Cruz, beta-Catenin (monoclonal, mouse) Abcam, CD 68 (polyclonal, rabbit) - Acris, c-Myc (polyclonal, rabbit) - Santa Cruz, Cox-2 (monoclonal, rabbit) - DCS Innovative Diagnostic Systems, ESDN (polyclonal, rabbit) - MoBiTec, Gas6 (polyclonal, goat) - Santa Cruz, Ki67 (monoclonal, mouse) - Dako, MMP2 (polyclonal, rabbit) - Biomol, Notch-3 (polyclonal, rabbit) - Santa Cruz, p53 (polyclonal, rabbit) - Santa Cruz, S100 (polyclonal, rabbit) - Abcam, SMA (polyclonal, rabbit) - Dako, TGF-beta (polyclonal, rabbit) - Santa Cruz, TNF-R2 (polyclonal, goat) - Santa Cruz; TUNEL- kit for apoptosis (APOPTAG^®^) - Chemicon. Secondary antibody anti-goat (rabbit) - Dako, anti-mouse (rabbit) - Dako. Examples for positive staining pattern of each marker are shown in Additional file 3, **Figure S3**.

### Statistical analysis

All together we analyzed 20 variables. Beside time and therapy we included 16 protein expression markers, and two collagen related parameters (collagen type I, collagen type III in scar).

For comparing expression levels of sole marker proteins we performed a multivariate ANOVA (SPSS 17.0 for Windows), and in case of significant differences followed by post hoc Bonferroni. A p of < 0.05 was considered as significant.

Because high correlations reflect similar intensity of expression even in case of considerably varying expression levels, Spearman's correlation coefficient was calculated. Whereas in absence of any functional relationship all correlation coefficients should be of about zero, we expected correlation coefficients of up to -1 or +1 in case of close functional relationship. Each of 20 variables can be related to 19 partners with 19 corresponding correlation coefficients. Altogether, for the entire data set there were 380 possible correlation coefficients, which, by ignoring the order (correlation AB = BA), can be reduced by half to 190 different correlation coefficients. Significance of the correlation coefficient was tested with Student's t-distribution and accepted if p < 0.05.

To estimate, how many significant correlation coefficients may be expected by chance, we used as control a simulation of a matrix of 20 variables with each 50 cases given by random numbers (in the range of 0 to 3, adapted to scores of histochemistry). Multivariate analysis as well as analysis of the correlation coefficients was done as described above.

## Results

The clinical course of all animals was uneventful; in particular the skin did not show any inflammation or sign of atrophy.

### Multivariate analysis for the impact of GC

All immunhistochemical markers showed at least some cells with positive staining, however, with considerable variation between animals and areas of interest (Additional file 4, **Table S1**). In general, highest expression scores were seen for beta-Catenin followed by Notch-3. Other proteins with an intense expression in at least 30% of the cells were c-Myc, MMP-2, TGF-beta, S100 and COX-2.

Local application of GC modified the composition of collagens in scar tissue and changed the expression of most molecular markers investigated.

**Figure 1:**
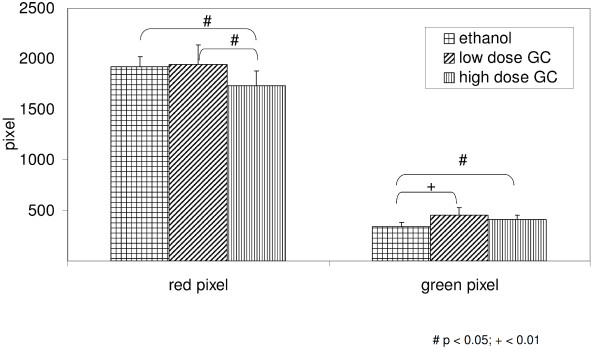
**Collagen type I (red pixel) and collagen type III (green pixel) in skin scar (number of pixel) after treatment with ethanol, low dose GC and high dose GC for 1 to 3 weeks (# p < 0.05; + p < 0.01)**.

Collagen type I amount was decreased in scar after Hd-GC (Hd-GC vs. ethanol: p < 0.05). Inversely, collagen type III was significantly increased following any GC treatment (Figure 1).

Separate analysis for the 6 areas of interest (as stated in Additional file 4, table S1 and Additional file 5, table S2) showed that, over all, GC significantly affected protein expression, but the impact on the various marker proteins differed markedly. Whereas multivariate analysis of the infiltrate for comparing the different time points revealed an independent impact only on TNF-R2, the comparison of treatment (ethanol, Ld-GC and Hd-GC) showed a significant impact of therapy on ESDN, MMP-2, TGF-beta, apoptosis, CD 68, TNF-R2, SMA und S100.

### Impact of GC by Spearman's correlation coefficients

For all 20 markers, there were 68 significant correlations, making 17.9% of the possible 380 correlations. The mean Spearman's correlation coefficient in the infiltrate was almost zero (-0.006) with a standard deviation of 0.267. Every significant effect of GC indicated by multivariate analysis was confirmed by significant Spearman's correlations coefficients. (Even if the analysis was performed separately for the various layers, almost all significant correlation coefficients were confirmed by multivariate analysis; Additional file 5, **Table S2**)

Whereas TGF-beta, TNF-R2 and MMP-2 were related to therapy with positive correlation coefficients (means an increased expression with higher GC concentration), the other five markers (CD68, apoptosis, collagen type I scar, ESDN, and S100) showed a negative correlation coefficient (less expression with higher GC concentration; Table 1).

**Table 1:** Depiction of significant correlation coefficients between markers and therapy in the infiltrate of the scar

	Spearman's correlation coefficient
CD68	- 0.730
Apoptose	- 0.723
Collagen type I scar (red pixel)	- 0.538
ESDN	- 0.408
S100	- 0.401
TGF-beta	+ 0.407
TNF-R	+ 0.566
MMP-2	+ 0.589

### Depiction of collaborative network and comparison with *in silico *simulation

**Figure 2:**
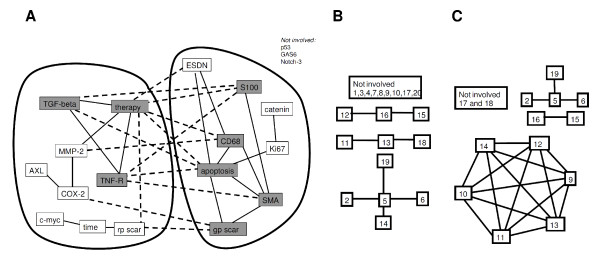
**Correlation networks and their analysis in a fictive model system**. **2A: **Depiction of the collaborative network in the cell infiltrate using significant Spearman's correlation coefficients (p < 0.05). Highly connected proteins with more than 3 significant correlations are marked in grey color; solid lines mark positive correlation coefficient, dotted line negative correlation coefficient. The analyzed tissue proteins include TGF-beta, MMP-2, TNF-R2, COX-2, AXL, c-myc, Ki67, beta-Catenin, ESDN, p53, Gas6, Notch 3, CD68, apoptosis -TUNEL, SMA, collagen type I - red pixel and type III - green pixel. Three therapy groups were compared; ethanol-controls, low dose GC and high dose GC. **Figure 2B: **Depiction of significant Spearman's correlations within a database of 20 variables, each consisting of 30 random data sets. **Figure 2C: **Depiction of significant Spearman's correlations within a fictive random database of 20 variables after introduction of a cluster, which included the variables 9 to 14 with equal numerical values, and which led to correlation coefficients of r = 1 between these variables.

In total, 16 of the 20 markers could be connected by significant correlation coefficients (Figure 2A). The cross-linked markers could be arranged in 2 clusters containing several positive correlation coefficients. One included TGF-beta, TNF-R2 and therapy as highly cross-linked centers (more than 3 significant correlations), the other S100, SMA, CD68, apoptosis and collagen type III. These two clusters were connected by several negative correlations, only.

To exclude that these significant results should be regarded as statistical effect of many comparisons, we repeated the analysis with a model of random figures. The multivariate analysis of this random data base (mean 1.46 +/- 0.85) did not reveal any significant impact of any of the variables at all. However, the analysis of the correlation coefficients for the random data matrix of 20 variables resulted in 16 significant correlation coefficients (4.2% of all), linking 11 variables to each other. Interestingly, these were only connected in a linear manner without any circular cross-linking (Figure 2B). The mean value of all 380 possible correlation coefficients was close to zero (0.018), with a standard deviation of 0.173. The introduction of a cross-linked cluster into the random database by using similar values for the variables 9 to 14 led to correlation coefficients of r = 1 between these variables (Figure 2C). However, none of the other variables were attached to this cluster.

**Figure 3:**
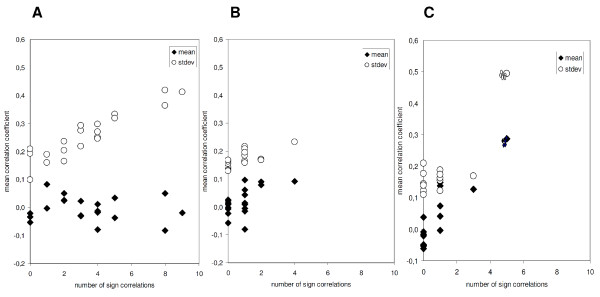
**Number of correlation coefficients with significance in relation to mean and standard deviation of all coefficients per variable**. **3A: **20 markers of the cell infiltrate underneath the scar with mean and standard deviation of all 19 correlation coefficients of every variable in relation to the number of significant correlations per variable. A standard deviation of more than 0.2 indicated highly cross-linked marker proteins, whereas the mean of all coefficients did not. **3B: **20 variables in a fictive model of random figures with mean and standard deviation of all 19 correlation coefficients of every variable in relation to the number of significant correlations per variable. With means between +0.1 and -0.1 and standard deviations of less than 0.25 highly cross-linked variables were rare. **3C: **20 variables in a fictive model of random figures after introduction of a cross-linked cluster with mean and standard deviation of all 19 correlation coefficients of every variable in relation to the number of significant correlations per variable. A standard deviation of more than 0.2 indicated highly cross-linked variables.

Both, in the biological network as well as in the model, there were some variables, which were highly linked to others. These variables were characterized by a pronounced variation of all correlation coefficients of this variable resulting in a higher standard deviation. Correspondingly, both in the model as well as in the biological system, a standard deviation of more than 0.2 strongly indicated a highly cross-linked variable with more than 4 significant correlation coefficients (Figure 3A-C). In contrast to the *in silico *modeling with the intrinsic cluster, these highly linked markers were not characterized by an increased mean of the correlation coefficients in the biological system.

## Discussion

The main findings of our experimental data describe glucocorticoids (GC) with its differentiated effects on the protein expression of the skin. Application of different doses of GC altered the investigated set of proteins specifically. With a novel approach of analysis we describe the pattern of GC-affected proteins as an interactive network, which was specifically changed in dependency of GC- dose and time.

First described as hormones produced by the adrenal glands, GC are extremely powerful substances, prescribed mainly for the management of inflammatory diseases. However, treatment with GC often causes additional and considerable side effects. E.g., the use of GC after surgical intervention has been associated with impairing the process of wound healing [6]. Uncertainty exists, whether this is a consequence of the anti-inflammatory effect, or a direct interference with formation of extracellular matrix, inclusive collagen as its main component, or both.

During tissue remodeling multiple proteins are involved, which reflect cellular signaling with a shorter half-life than collagen; we choose some of them and analyzed all together 16 markers of cell-physiology in different layers of skin and scar. As part of the inflammatory system we selected CD68, AXL, Gas6, TNF-R2, COX-2, S100, as regulator of the extracellular matrix MMP-2, TGF-beta, p53, for the process of cell differentiation and regeneration SMA, beta-Catenin, Notch-3, c-Myc, ESDN, and finally Ki67 representing activity of proliferation and TUNEL as indicator of apoptosis.

We could demonstrate that therapy with GC changed both, the composition of collagens as well as the expression of many different marker proteins. Though we could illustrate the complex role of glucocorticoid treatment for every layer of skin, it was intriguing to see the considerable differences in the 6 layers, which were investigated separately. These complex changes have to be expected, considering the study of Maurer, who studied gene expression level in cultured cells and showed that GC were able to regulate 2461 of 4943 genes. 1982 of these genes were only altered by dexamethason, the remaining ones were co-effected by testosterone and/or DHEA as well [7].

The usual statistical analysis from immunohistochemical results is based on the comparison of means or medians between different groups. Accordingly, it is assumed that any treatment with GC may result in a change of mean expression level of any of the markers. However, comparing mean expression levels may neglect the dynamic character of a tissue compound, reflected by the considerable variation of expression levels between different tissues, layers or species. Instead of a constant increased or decreased expression of any marker after an intervention, a subsequent regulatory process is initiated, which may even counteract the initial process, and thereby often reduce any deviation of mean or median expression. In this regard, most biological processes are not only part of a network, but furthermore part of biological systems with coherent collective dynamics formed by numerous feed-back loops for its regulation, which are insufficiently characterized by simple and rigid assessment, and not characterized by the presence of correlations between individual molecular reactions [8].

As part of a system, which tries to keep homeostasis by integration in feedback loops, the cells will change their expression level periodically. Accordingly, oscillations have been seen for cell growth in culture or for metabolites as lactate dehydrogenase and nicotinamide dehydrogenase [9-11]. Recently, similar oscillations have even been described on gene level [12]. These fluctuations over time have to be considered for all cybernetic systems, and in particular for biological networks. Whereas comparison of means is susceptible to the variations over time so that any effect easily is overlooked, it is the analysis of correlation coefficients, which is more resistant to periodical fluctuations and helps to find functional relations and its change.

Any therapy alters not only the absolute expression level but also the mode of interaction between two markers, eventually increasing or decreasing the correlation coefficients between two markers in dependency of the intervention. In case of very strong therapeutic agents it may be suspected that therapeutic intervention challenges the physiological correlation network to more simple structures abolishing regulatory relations among the markers but linking all markers to the agent. Correlation coefficients reflect functional relationship between 2 components, ranging from 0 in case of random influence with each other to 1, which means strict linkage in any case with identical values. A first attempt of a protein-interaction map in humans depicted over 70 000 interactions for around 6 200 proteins [13]. We could show that calculation of correlation coefficients permits a depiction of a map of close functional relationship by linking markers with significant correlation coefficients. Furthermore, for a specific variable a high variation of the coefficient to others strongly indicates that the variable is highly cross-linked and is likely to be centrally involved in a functional cluster. This first impression may indicate ways to identify crucial pathways, and may help to reveal the collaborative structure of complex networks.

We simultaneously measured the expression of several proteins of cell-function and extracellular matrix and were able to confirm several pathophysiological findings and assumptions. Analysis of collagens confirmed the dominance of mature collagen type I in the dermis, whereas in scar tissue the amount of collagen type III was significantly higher. Probably due to the long half time of collagen type I there was no significant change in unwounded dermis after treatment with GC for up to 4 weeks. In contrast, in scar with predominance of increased collagen synthesis GC lowered the amount of collagen type I. In scar tissue GC increased the amount of immature collagen type III. This may be surprising because GC are supposed to reduce the inflammatory stimulus and collagen III accompanies inflammatory response in early wound response [14]. An increased amount of collagen type III in scar may indicate either an enhanced synthesis or a delayed degradation of collagen type III. It is reasonable that any disturbed maturation of collagen will be more apparent in newly formed scar collagen than during slow remodeling of unwounded skin. Overall, GC led to a decreased collagen type I/III ratio in scar with relative enhancement of collagen type III.

Furthermore, GC changed the expression of several marker proteins, as MMP-2, beta-Catenin, COX-2, ESDN, S100, SMA, Ki67, TGF-beta, TNF-R2, CD68 and c-Myc. They are all known to be involved in wound healing and tissue remodeling. For most of them an influence of GC has already been described. E.g. c-Myc and beta-Catenin act on WNT-pathway, thereby altering proliferation and differentiation, resulting in a chronic wound [15]. Furthermore, GC are known to increase the expression and act on MMPs, JAG1 (a Notch ligand), growth factors like TGF-beta, proliferation, tissue remodeling, angiogenesis, and apoptosis [16]. One of the major contributors to block inflammatory cascades is the negative regulation of crucial transcription factors, which are in the centre of many pathways, like nuclear factor-kappa B, the activity of the latter has been shown to be regulated by GCs, too [17].

We are aware of the limitations of this study, which include its basis on immunhistochemical analysis of the expression of proteins in tissues, which is only semi-quantitative. However, due to the limitations of any immunohistochemical analysis of tissues, a more exact characterization of protein expression in different compartments with the available tools is hardly possible. In addition, the observed variance between tissues and neighboring cells raises the question whether this would be possible at all. Furthermore, the observed expression mainly depends on the quality of antibodies. The use of new developed agents will most likely result in modified correlations. In addition, the study was done in mice and therefore translation of the results into humans has to be done with caution. However, though the correlation networks between species probably differ, there is no doubt that we have to consider multiple effects of glucocorticoids in humans, among them their complex effect on wounding and scar formation. Thus, regardless the number or type of marker proteins or which cell, tissue or species is investigated, the principal problem for current research persists and is discussed in this study that is how to interpret our *in vivo *data, which are active in a dynamic network of numerous interferences and interactions. The major challenge for future modeling of real biological systems will be the identification of causal pathways and collaborative clusters by the information given from simultaneous measurements of genes and proteins.

## Competing interests

The authors declare that they have no competing interests.

## Authors' contributions

All authors participated in the design of the study. UK carried out immunohistochemical analysis, performed statistical evaluation, handled financing and helped drafting the manuscript. NF performed animal experiments and retrieved the material for analysis. AF analysed the data and drafted the manuscript. All authors read and approved the final manuscript.

## Supplementary Material

Additional file 1 **Figure S1: **Experimental Setting Click here for file

Additional file 2 **Figure S2: **Areas of interest as defined for standardised analysis of the biomarkers. Click here for file

Additional file 3 **Figure S3: **Examples for positive staining pattern of the biomarkers. Click here for file

Additional file 4 **Table S1: **Mean (standard deviation) expression for all samples, listed separately for the 6 areas of interest (n = 30), scored from 0 to 3 for 0 < 5%, 1 = 5-30%, 2 = 30-80%, 3 >80% positive cells. Click here for file

Additional file 5 **Table S2: **Calculation of Spearman's correlation coefficients revealed significant relations of several markers with therapy (positive r means increasing expression for hd-gc > ld-gc > ethanol, negative r means decreased expression for hd-gc > ld-gc > ethanol); * not confirmed by multivariate analysis, ## in case of significant impact in multivariate analysis, only. Click here for file
