# Supplementary figures and images for: Evaluation of the collaborative network of highly correlating skin proteins and its change following treatment with glucocorticoids
Source: Theor Biol Med Model. 2010 May 28;7:16. doi: 10.1186/1742-4682-7-16 (PMC2901312; doi:10.1186/1742-4682-7-16)

## I. series

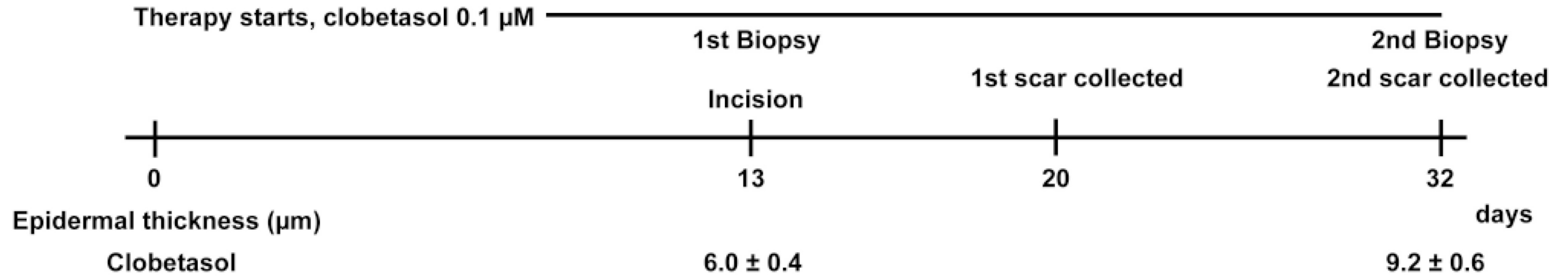

## II. series

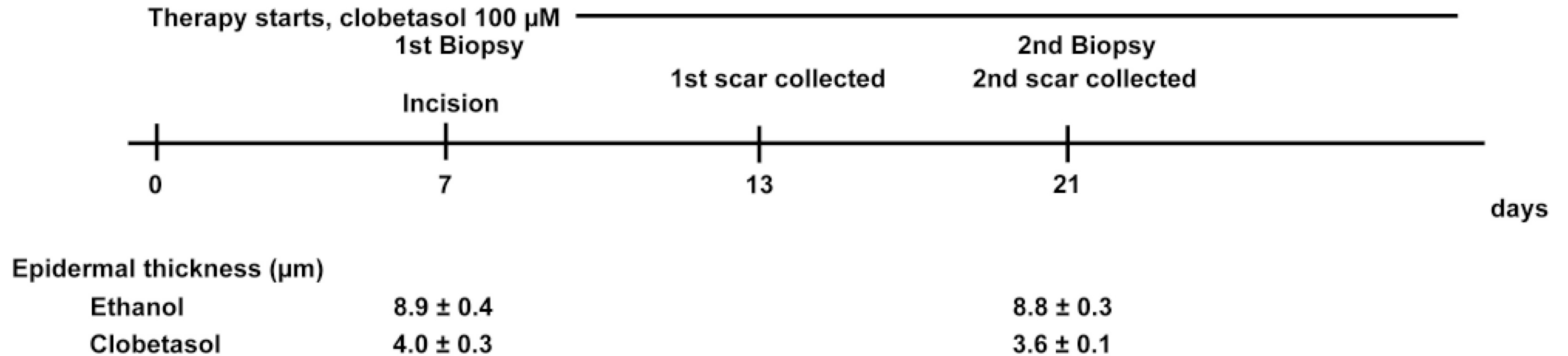

Supplement: Additional file 1 — Figure S1: Experimental Setting [file 1742-4682-7-16-S1.PDF]

Immunohistochemistry of skin and scar

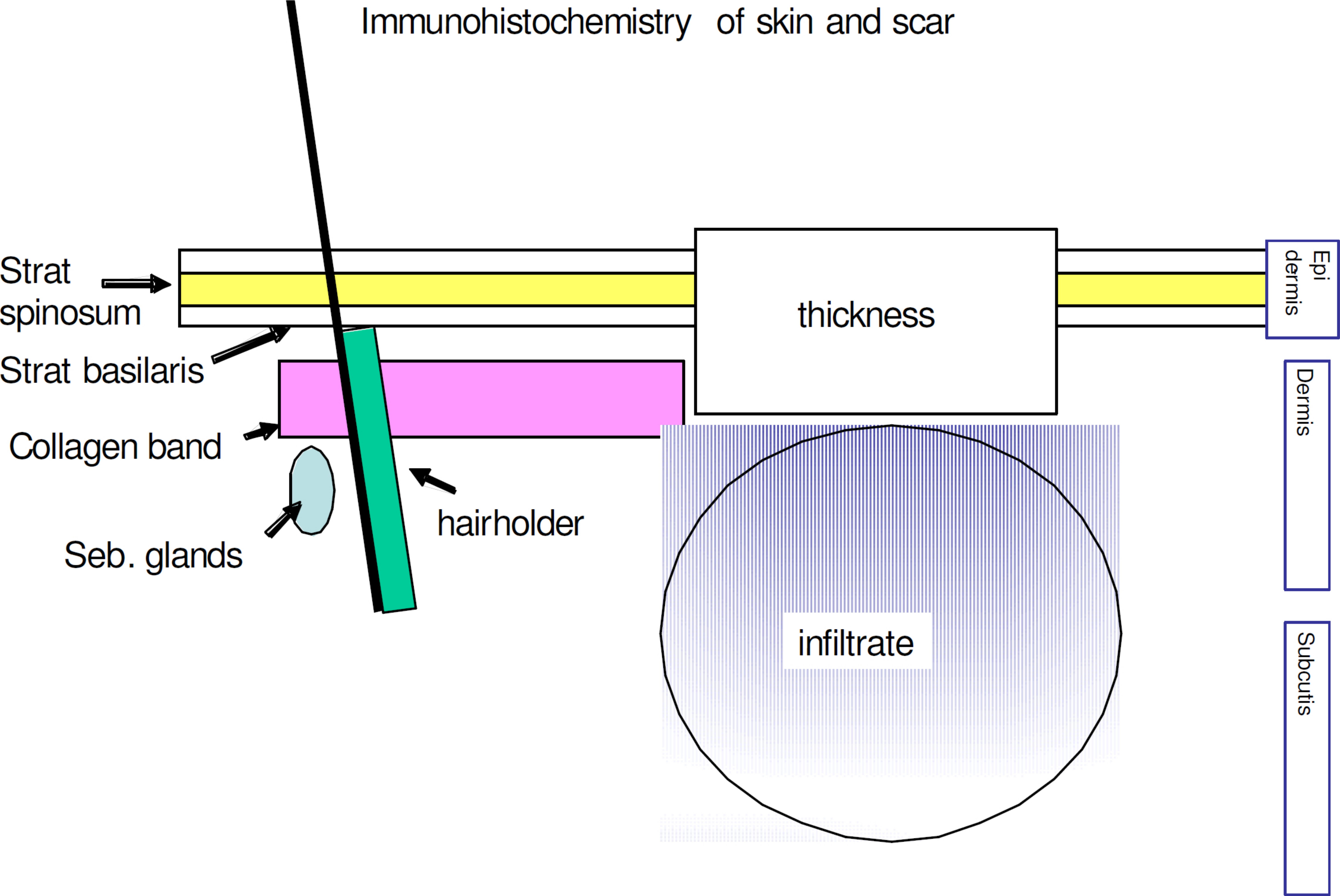

Supplement: Additional file 2 — Figure S2: Areas of interest as defined for standardised analysis of the biomarkers. [file 1742-4682-7-16-S2.PDF]

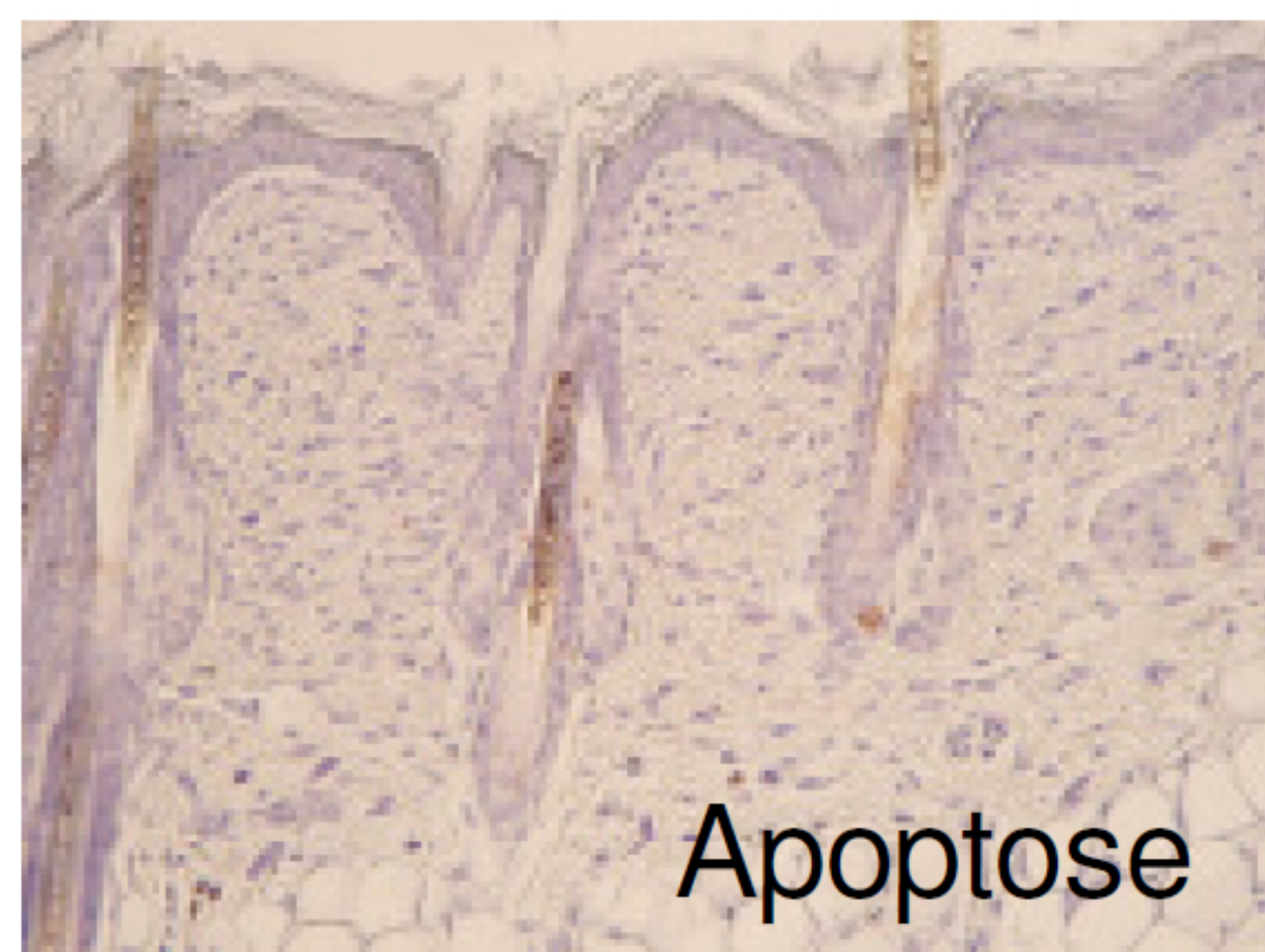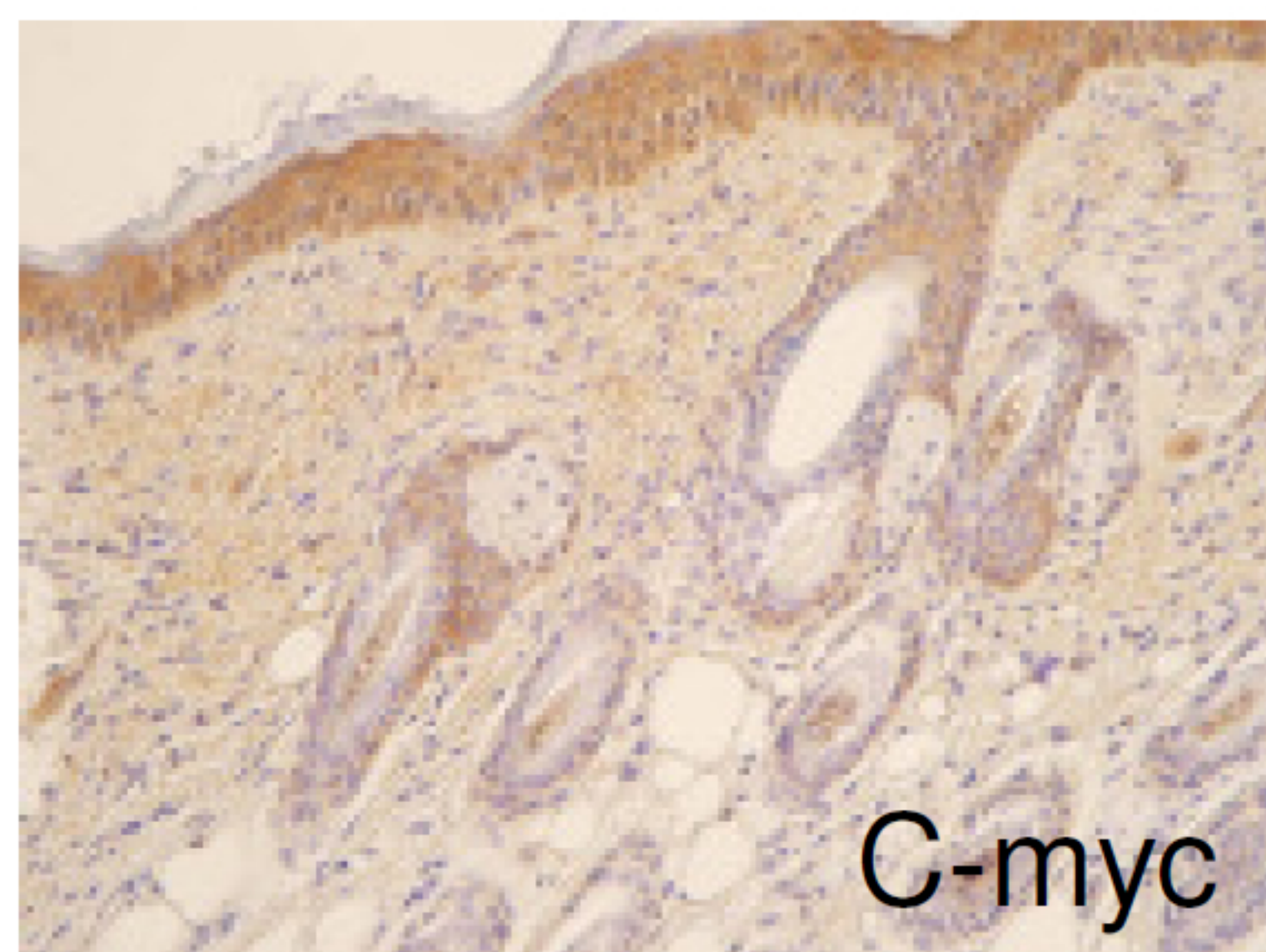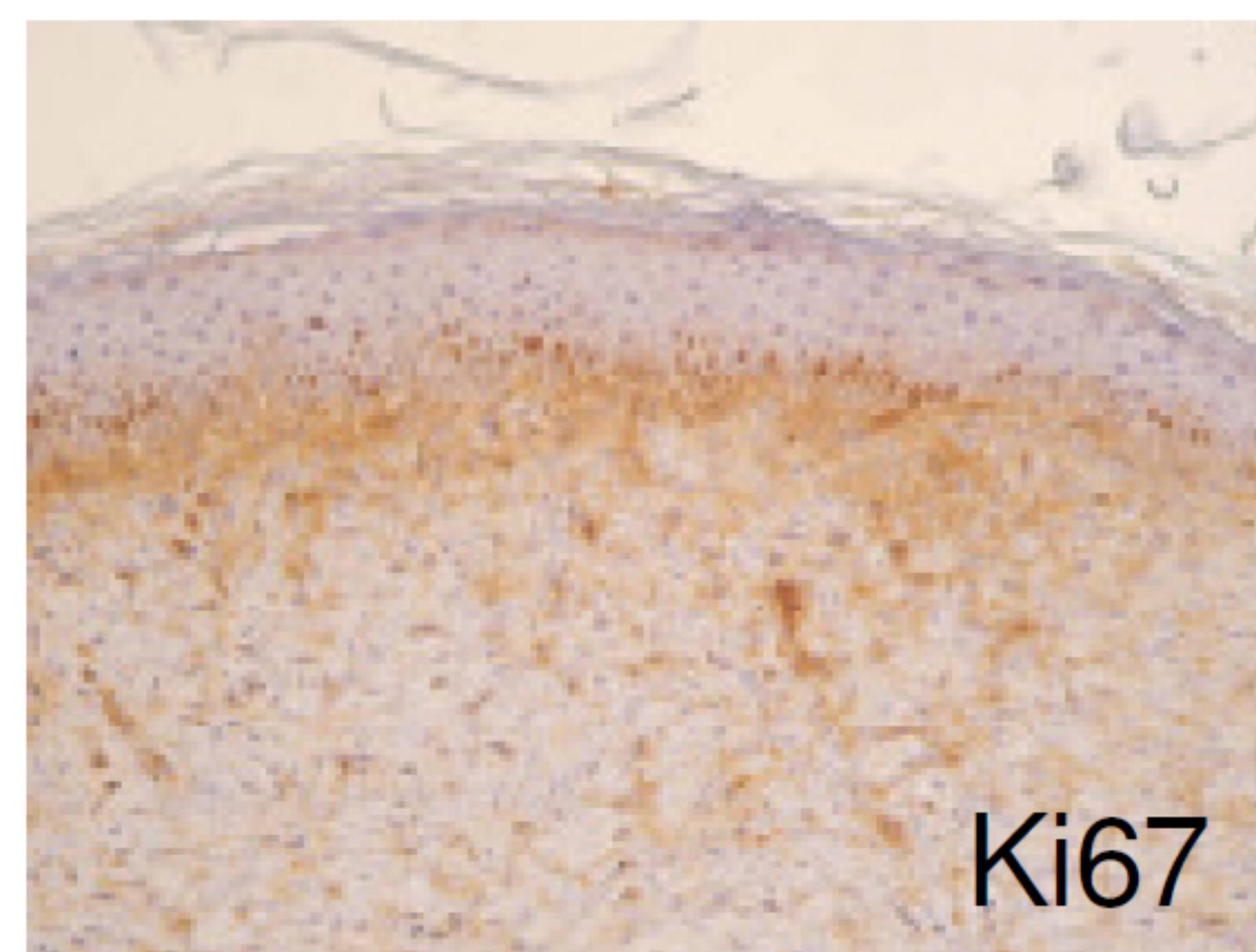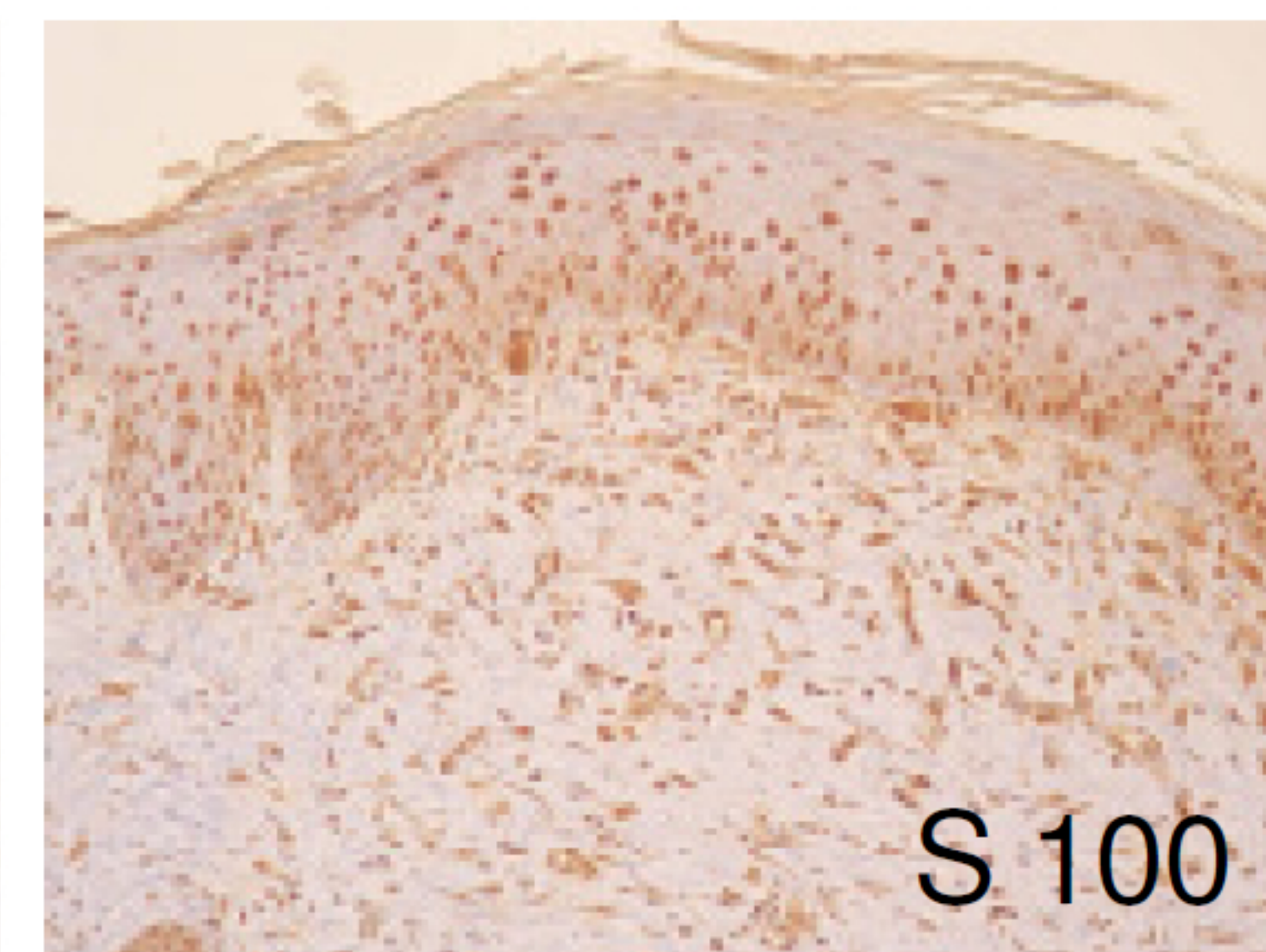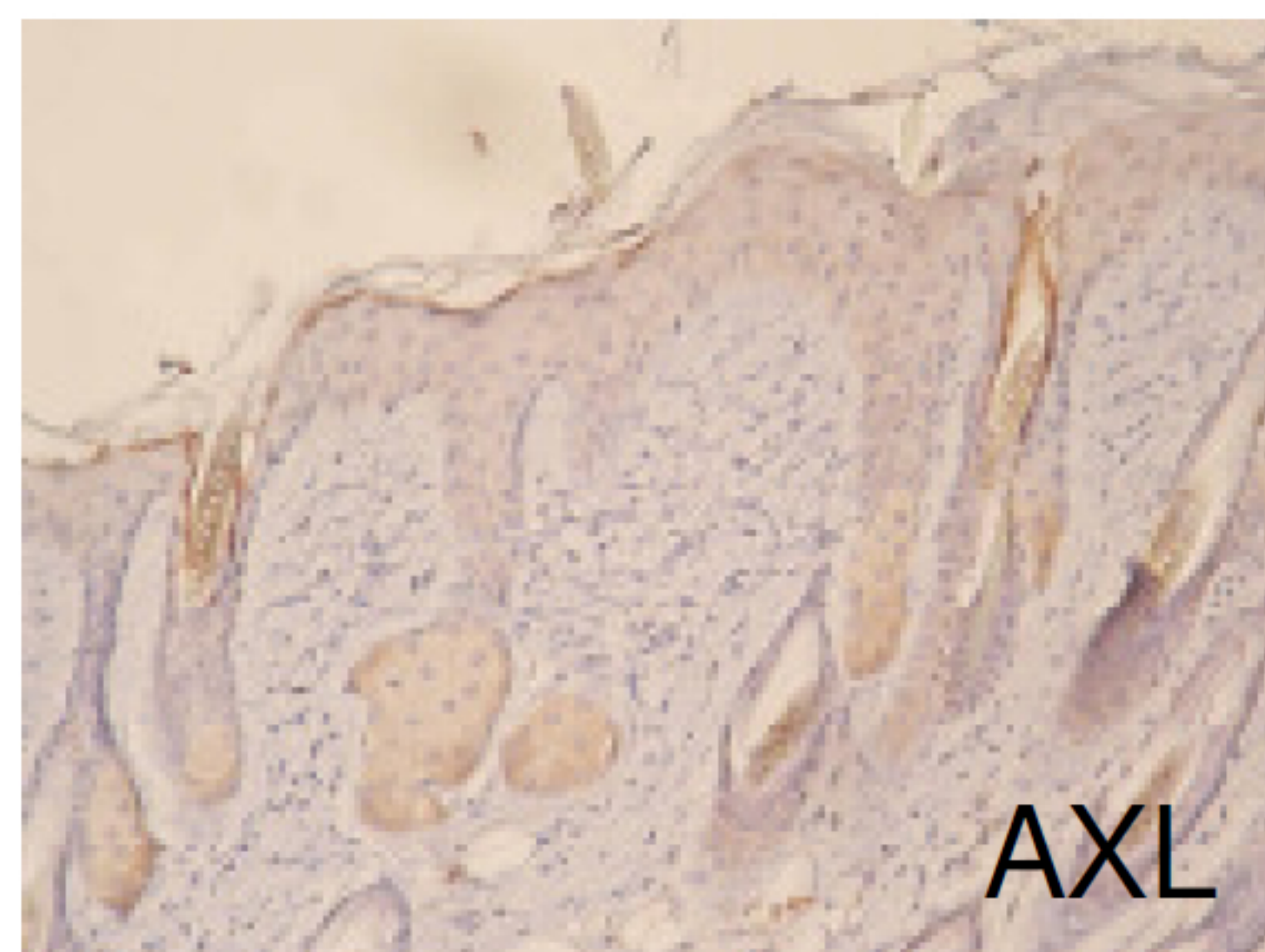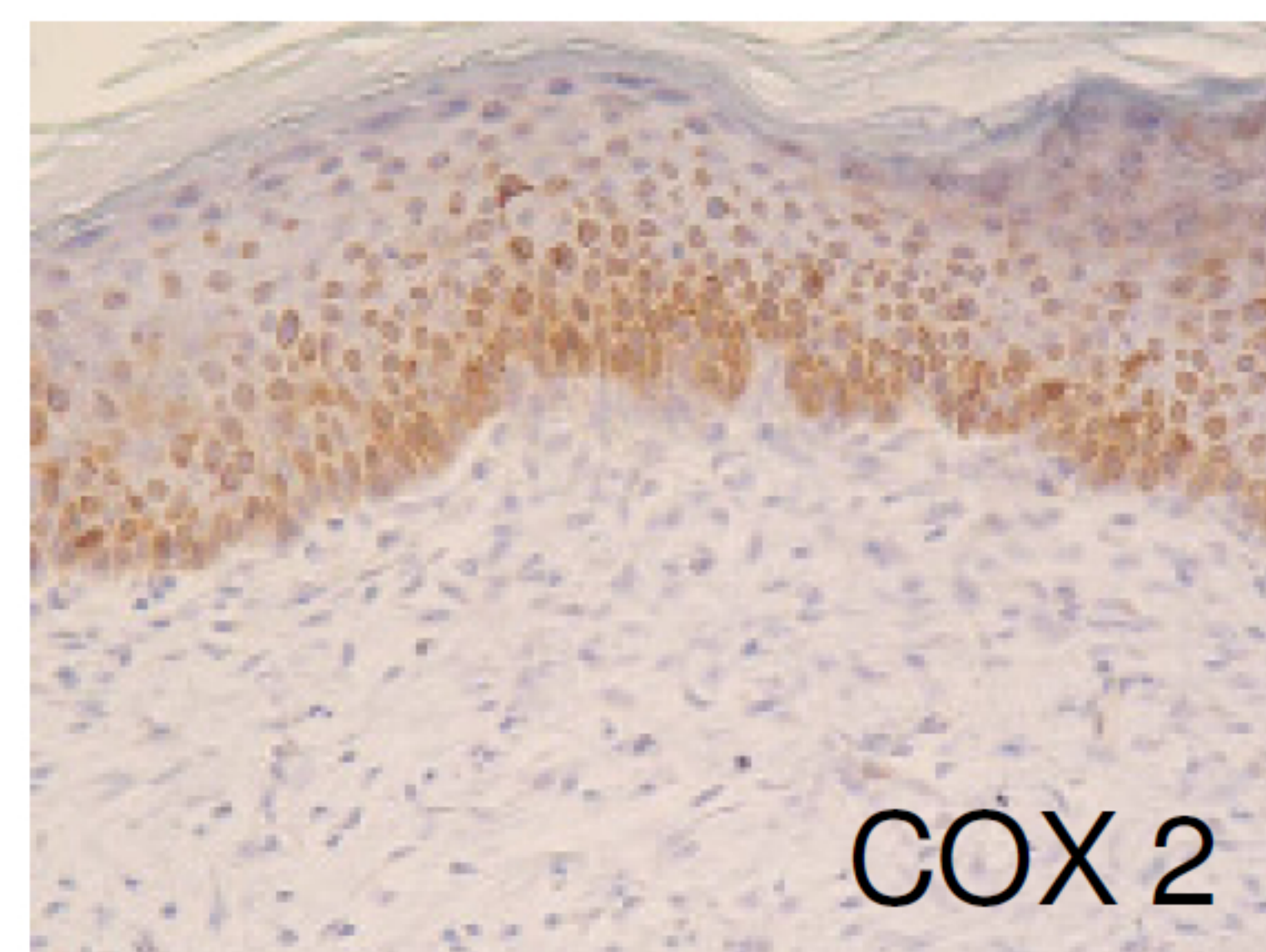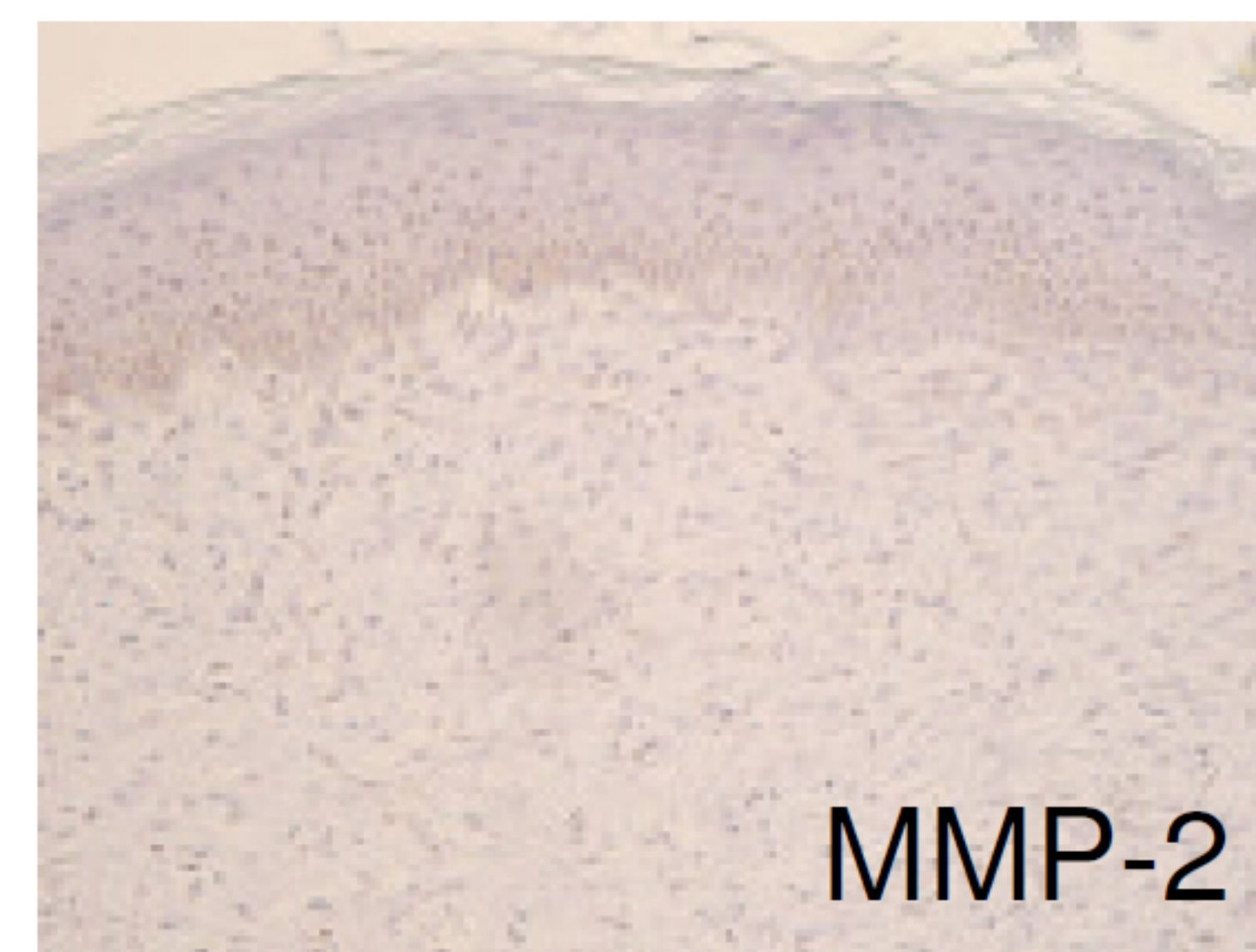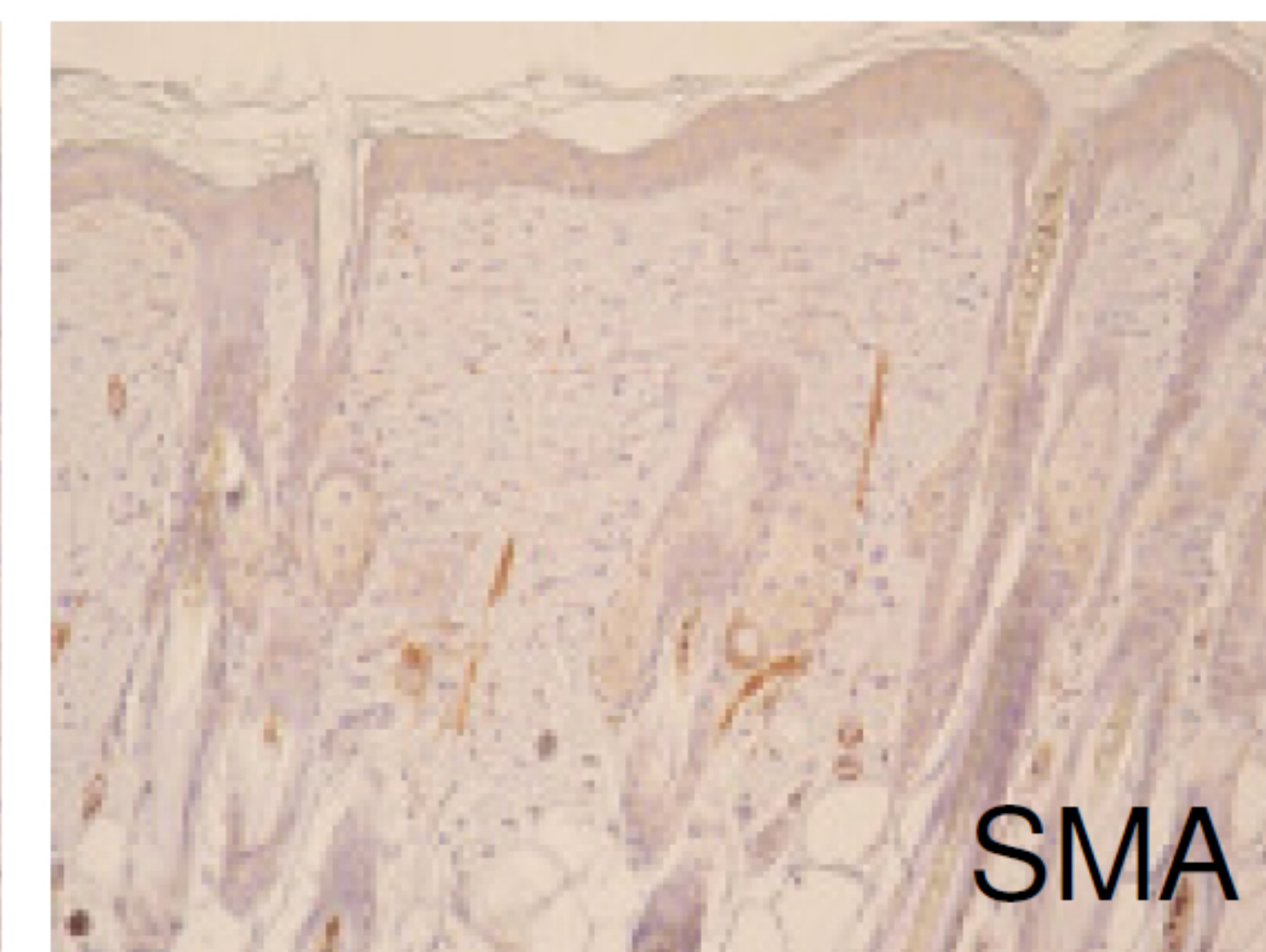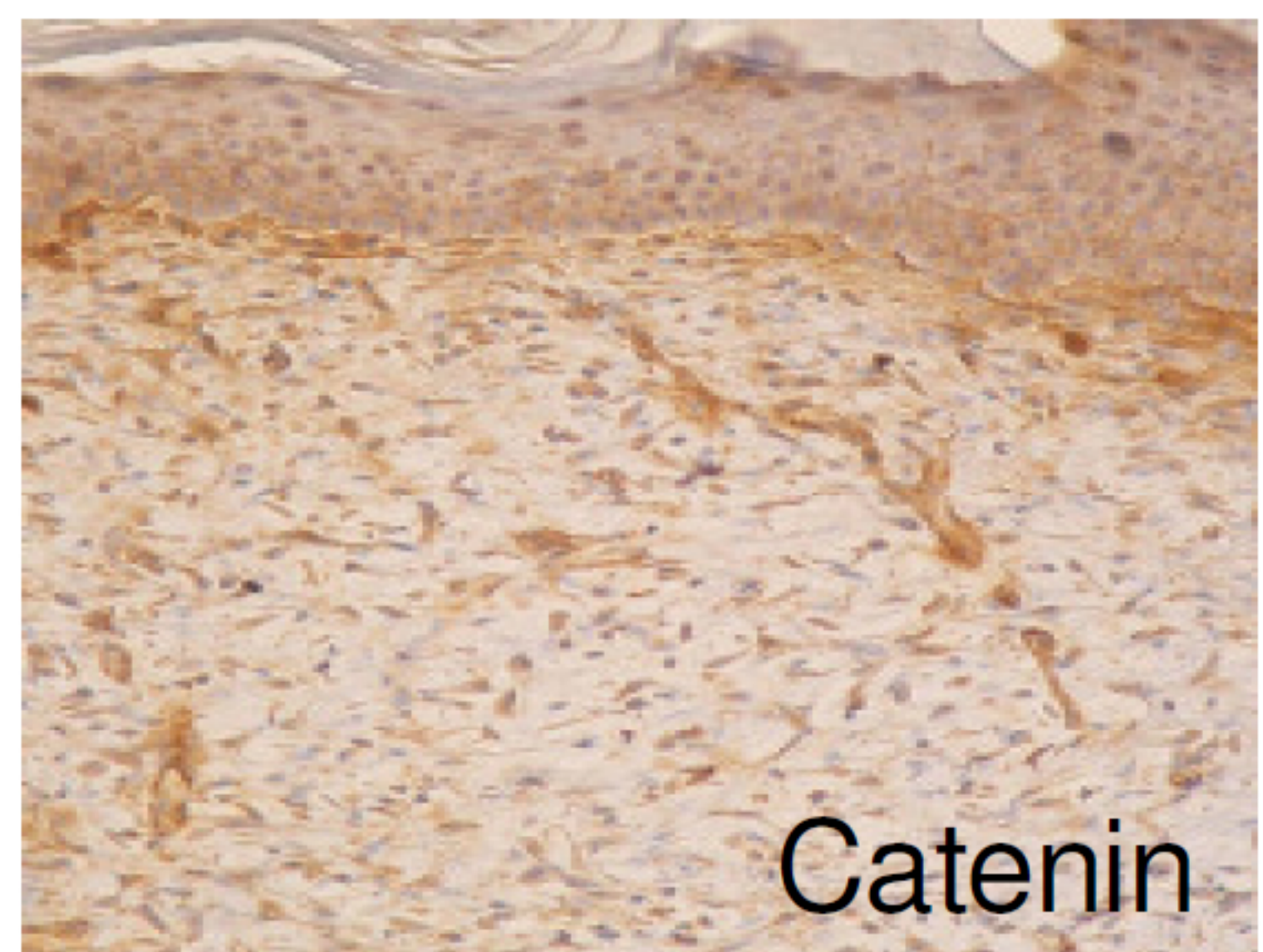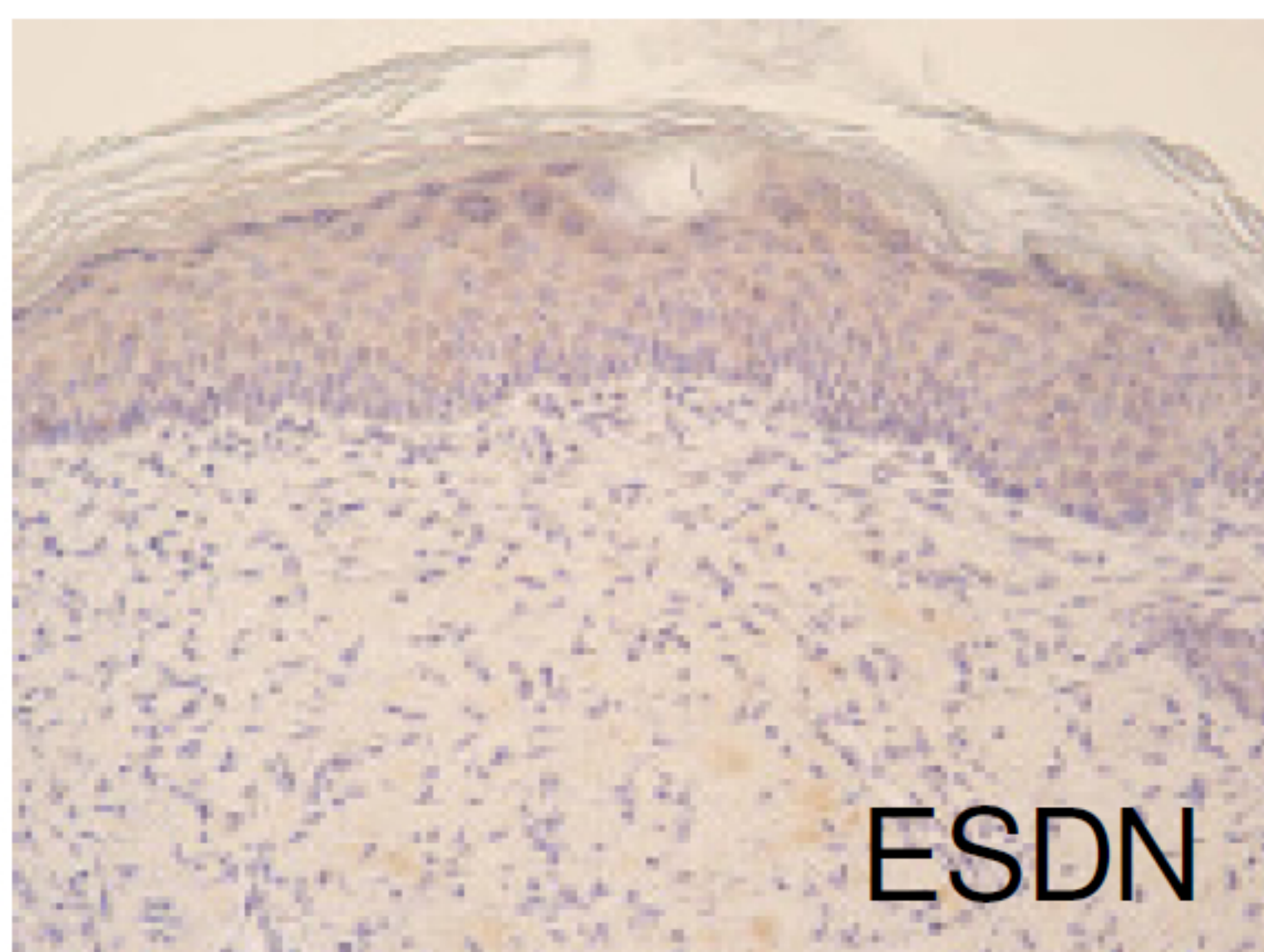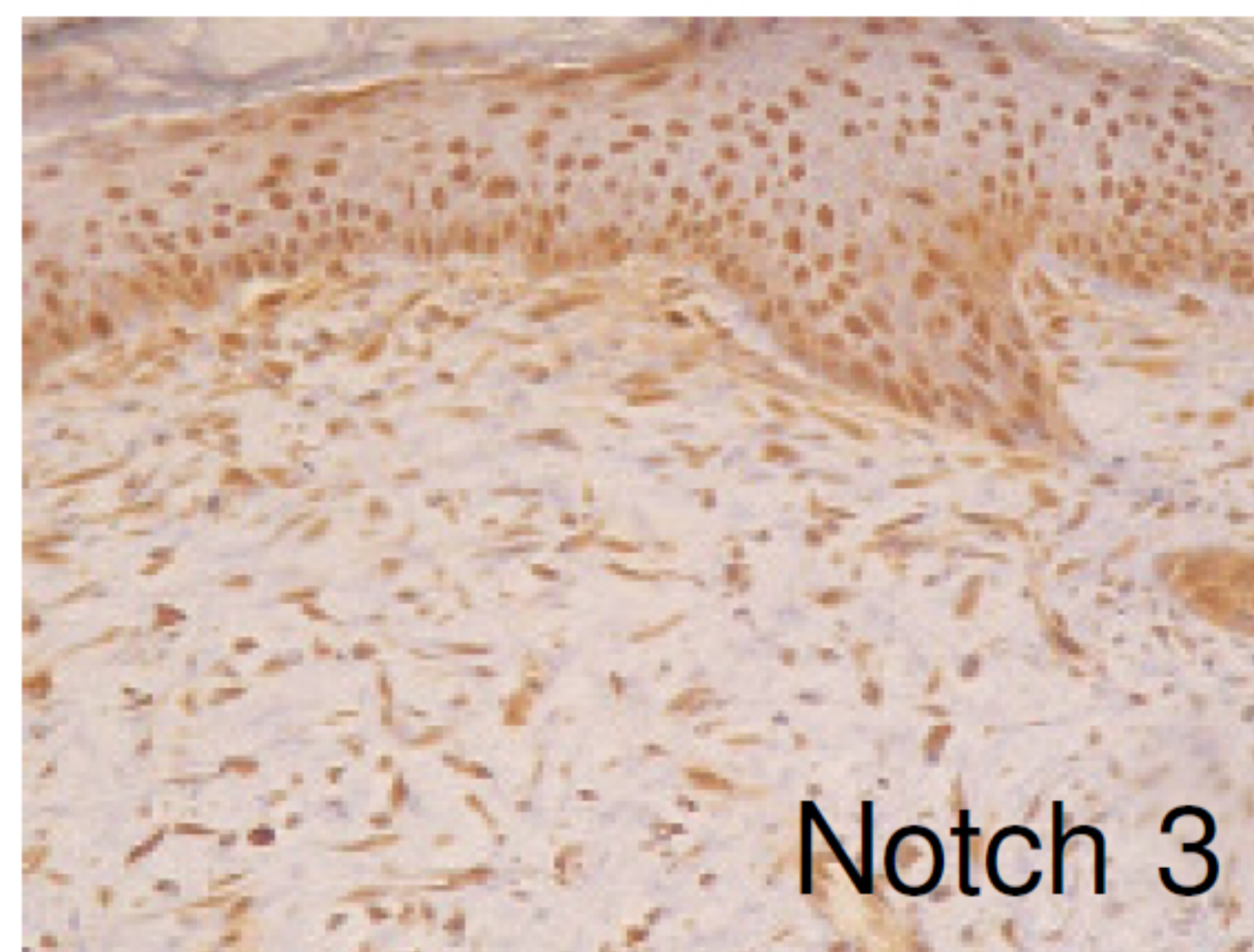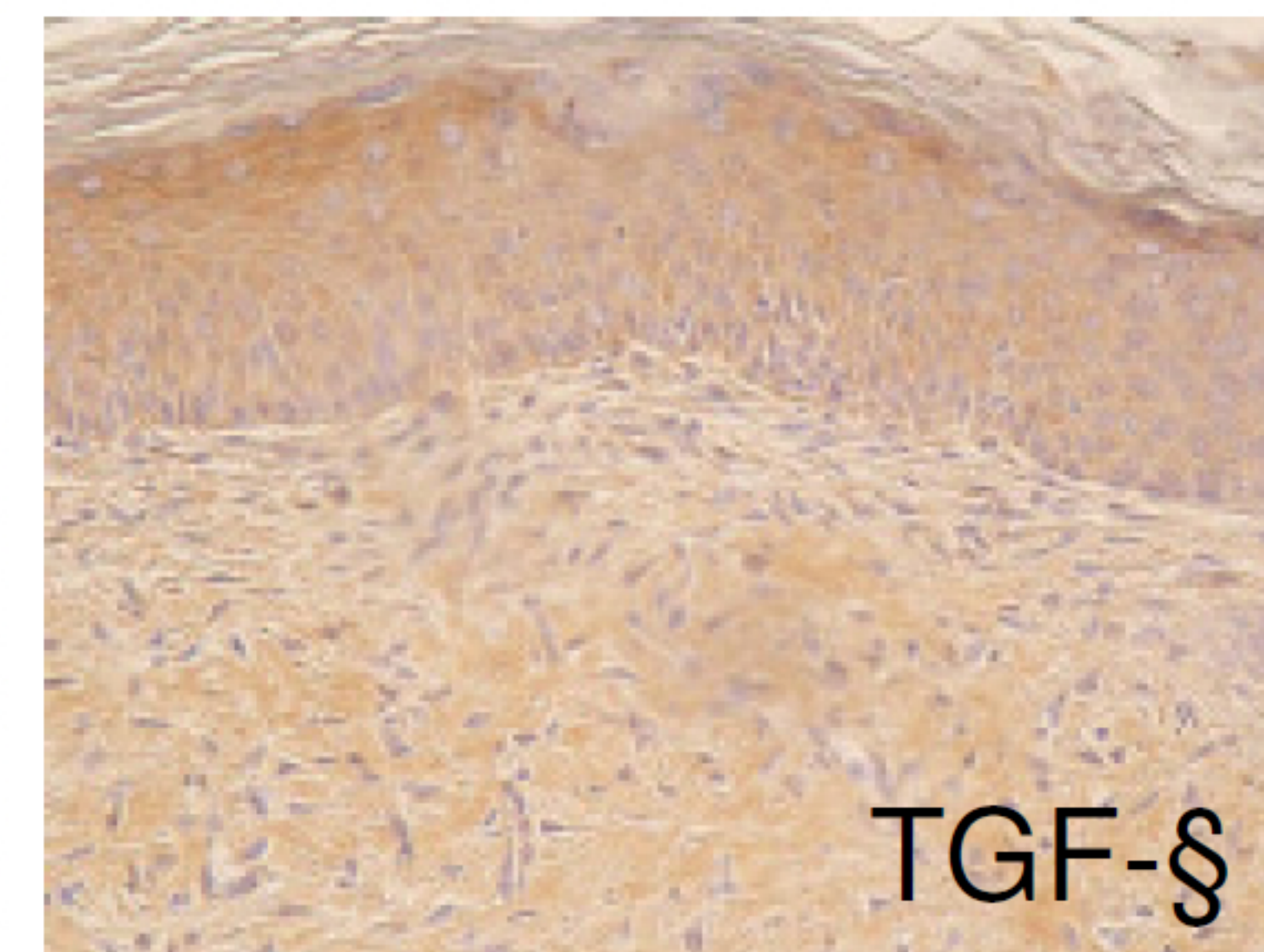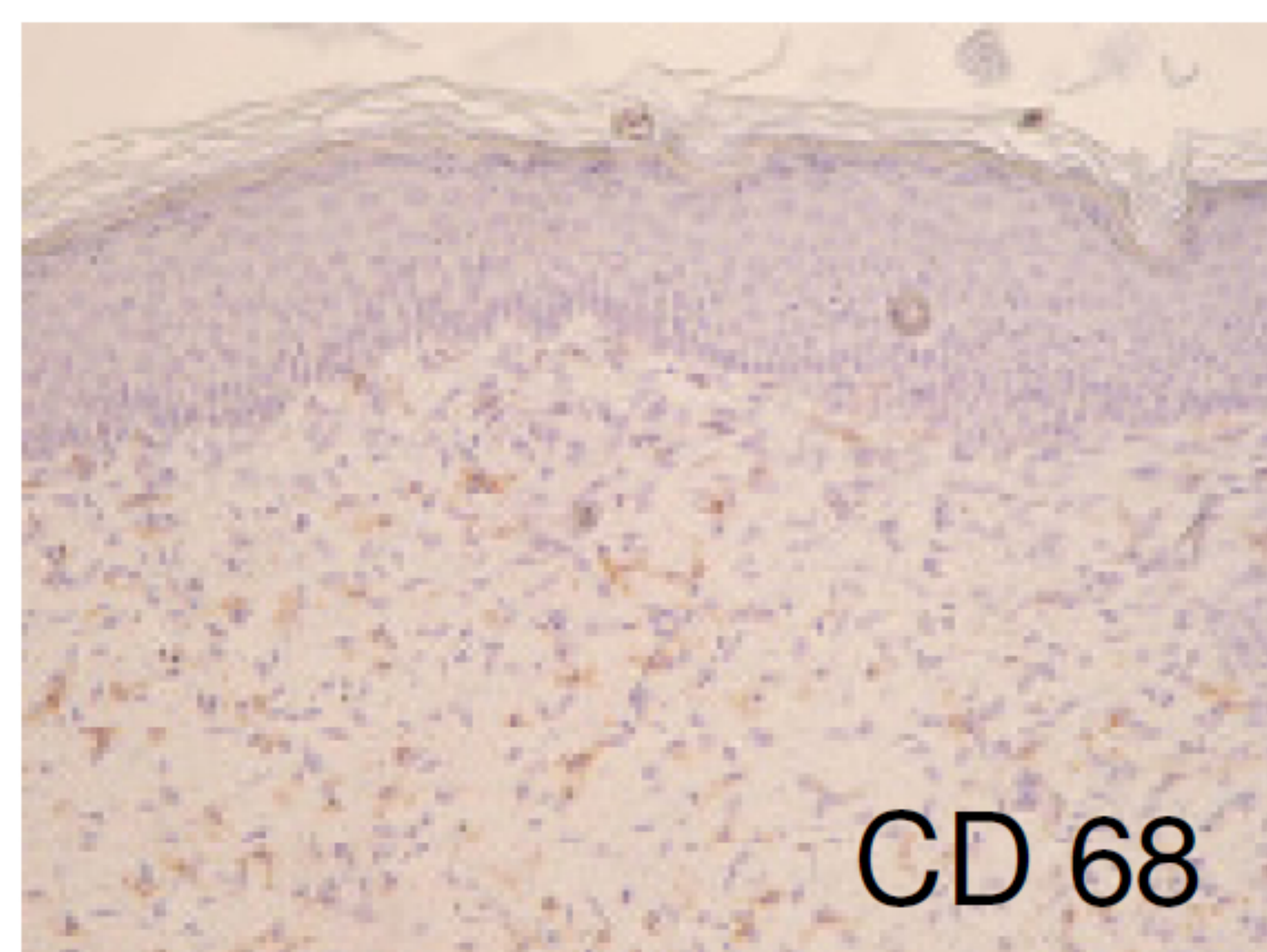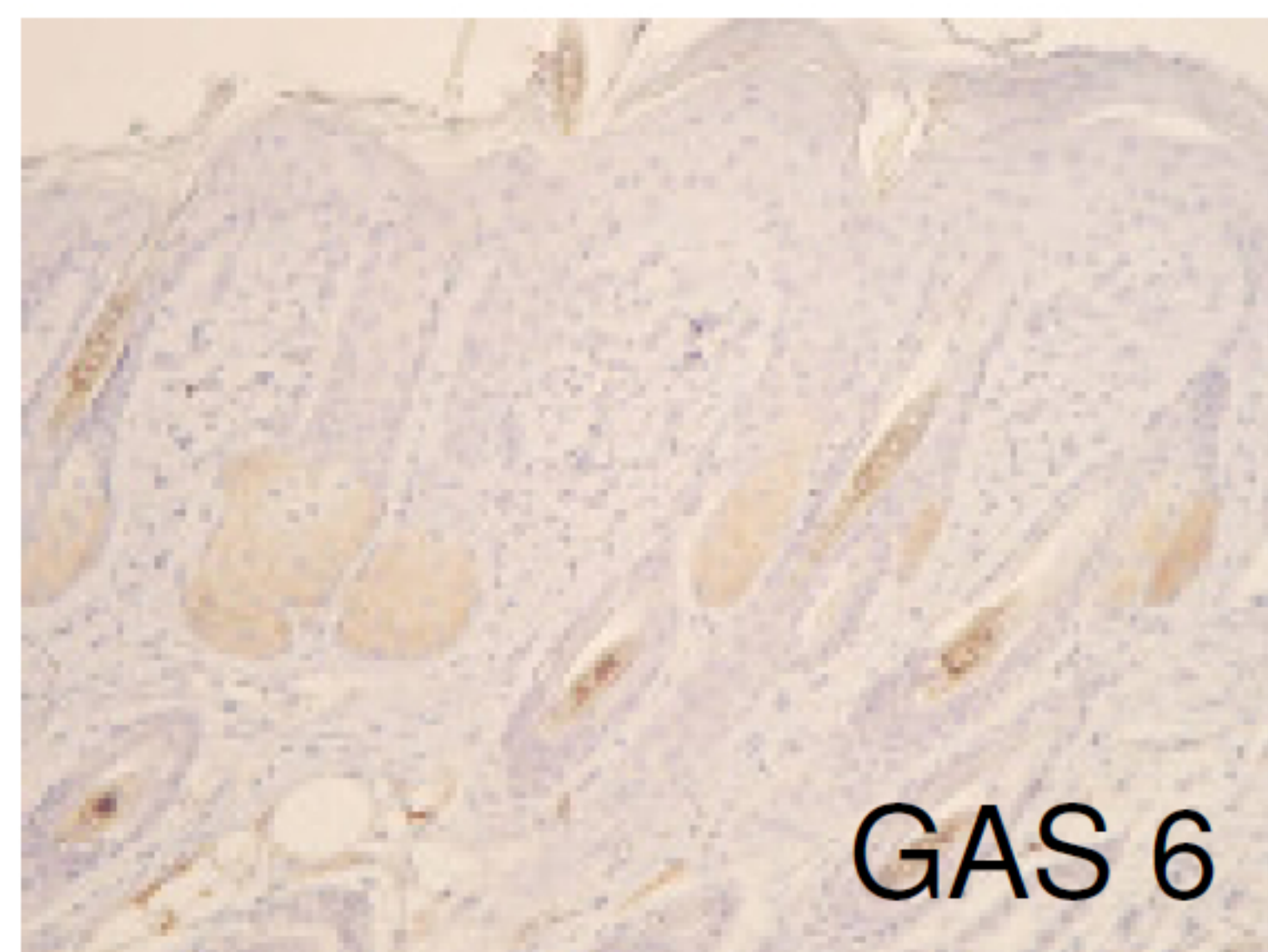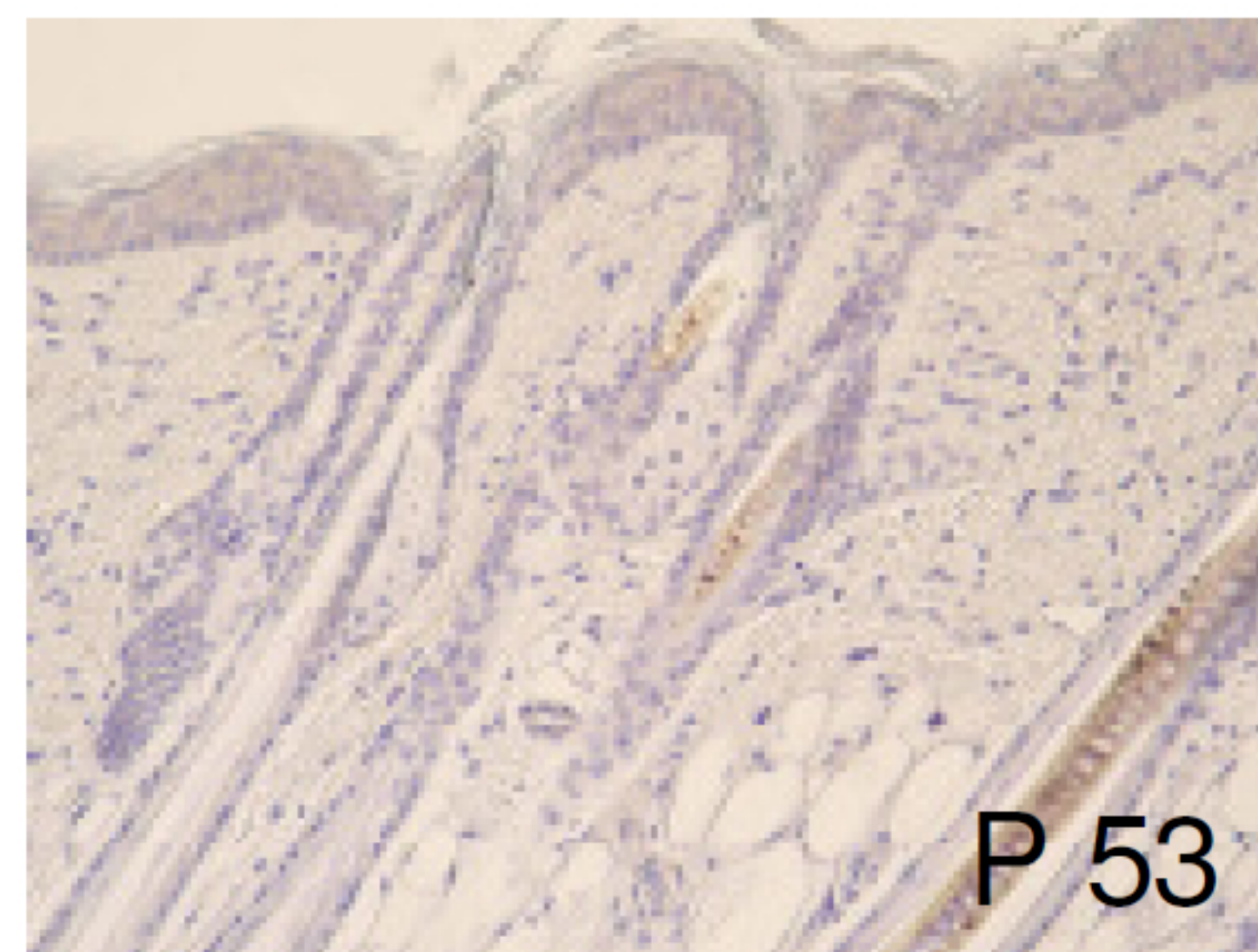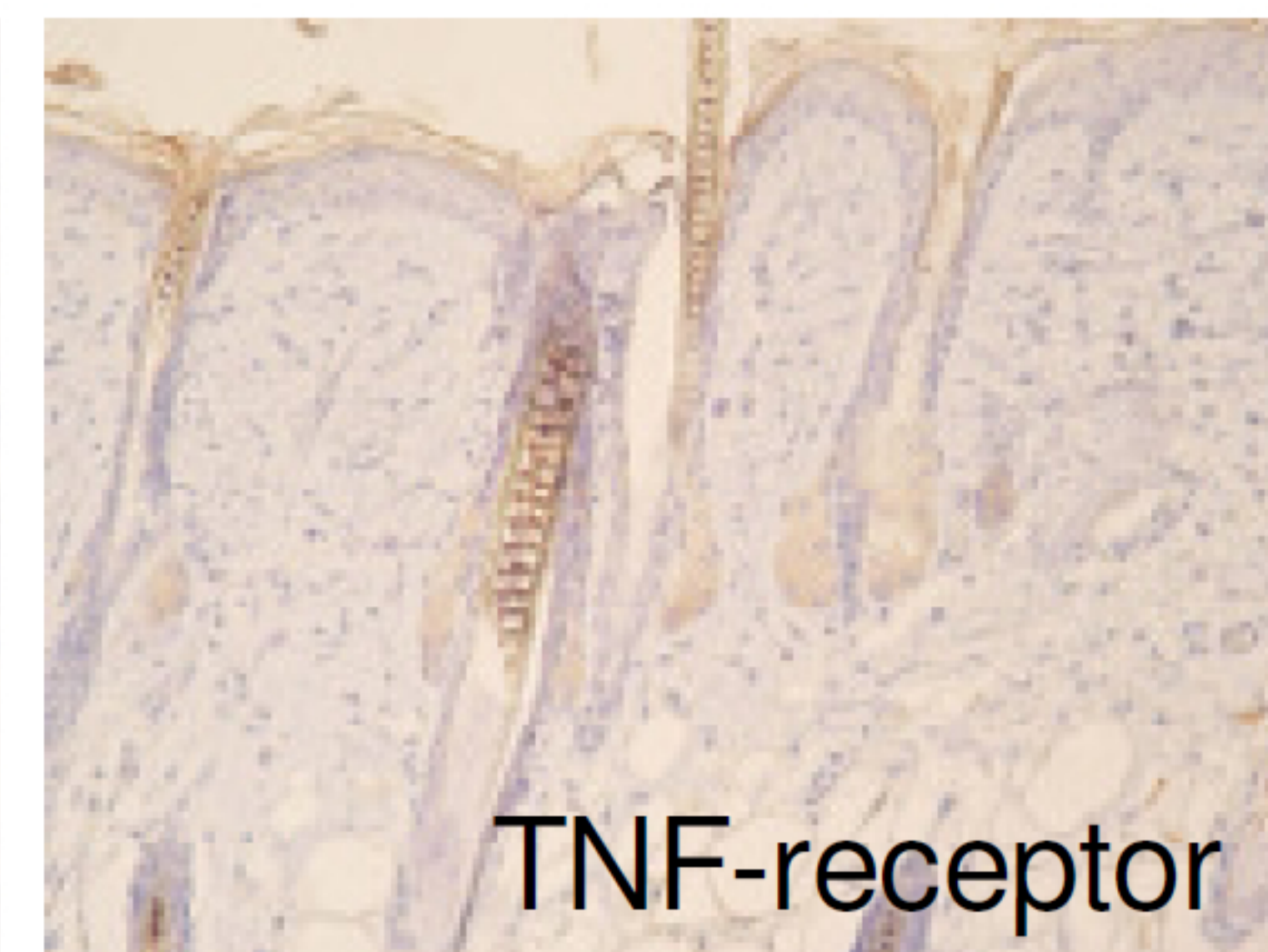

Supplement: Additional file 3 — Figure S3: Examples for positive staining pattern of the biomarkers. [file 1742-4682-7-16-S3.PDF]
