# Supplementary material for: Evaluation of the collaborative network of highly correlating skin proteins and its change following treatment with glucocorticoids
Source: Theor Biol Med Model. 2010 May 28;7:16. doi: 10.1186/1742-4682-7-16 (PMC2901312; doi:10.1186/1742-4682-7-16)
Supplement: Additional file 4 — Table S1: Mean (standard deviation) expression for all samples, listed separately for the 6 areas of interest (n = 30), scored from 0 to 3 for 0 < 5%, 1 = 5-30%, 2 = 30-80%, 3 >80% positive cells. [file 1742-4682-7-16-S4.DOC]

**Additional Table 1:** Mean (standard deviation) expression for all samples, separately for the 6 areas of interest (n=30), scored from 0 to 3 for 0=< 5%, 1=5-30%, 2=30-80%, 3=>80% of positive cells

|  |  | Stratum basale | Stratum spinosum | Seborrhoic gland | Hairholder | Infiltrate | Epidermal thickening |
| --- | --- | --- | --- | --- | --- | --- | --- |
| 1 | P53 | 0.30 (0.65) | 1.20 (0.89 | 0.20 (0.41) | 0.13 (0.35) | 0.97 (0.96) | 0.20 (0.48) |
| 2 | c-myc | 1.13 (0.97) | 2.33 (0.80) | 0.70 (0.84) | 0.80 (0.89) | 1.90 (0.85) | 1.10 (0.76) |
| 3 | ESDN | 0.07 (0.37) | 0.43 (0.86) | 0.10 (0.31) | 0.03 (0.18) | 0.43 (0.82) | 0.13 (0.43) |
| 4 | MMP-2 | 2.63 (0.62) | 2.53 (0.63) | 2.77 (0.43) | 1.83 (1.02) | 2.47 (0.57) | 2.33 (0.84) |
| 5 | Notch-3 | 2.60 (0.68) | 2.57 (0.68) | 2.80 (0.61) | 1.97 (0.89) | 2.63 (0.81) | 2.53 (0.73) |
| 6 | TGF-beta | 1.13 (0.82) | 2.27 (0.89) | 1.97 (0.89) | 0.60 (0.72) | 2.10 (0.85) | 1.17 (0.59) |
| 7 | Ki-67 | 1.93 (0.91) | 0.90 (0.80) | 1.90 (0.92) | 0.80 (0.85) | 1.67 (0.66) | 1.77 (0.73) |
| 8 | TUNEL | 0 (0) | 0 (0) | 1.67 (0.38) | 0 (0) | 0 (0) | 0.67 (0.66) |
| 9 | CD 68 | 0 (0) | 0 (0) | 0 (0) | 0 (0) | 0 (0) | 0.90 (0.85) |
| 10 | AXL | 0 (0) | 0.47 (0.68) | 2.00 (0.74) | 0.07 (0.25) | 0.57 (0.57) | 0.57 (0.73) |
| 11 | GAS6 | 0 (0) | 0(0) | 2.13 (0.78) | 0 (0) | 0.07 (0.25) | 0.13 (0.43) |
| 12 | TNF-R2 | 0.03 (0.18) | 0.07 (0.25) | 2.23 (0.63) | 0.03 (0.18) | 0.33 (0.45) | 0.97 (0.62) |
| 13 | SMA | 0.07 (0.37) | 0.43 (0.82) | 0.83 (1.09) | 0.17 (0.46) | 0.40 (0.86) | 0.47 (0.73) |
| 14 | S100 | 2.40 (0.62) | 2.13 (0.73) | 2.70 (0.47) | 1.50 (0.94) | 2.10 (0.85) | 2.40 (0.68) |
| 15 | Catenin | 2.63 (0.72) | 2.60 (0.68) | 2.80 (0.41) | 2.07 (0.87) | 2.50 (0.63 | 2.27 (0.64) |
| 16 | Cox-2 | 2.60 (0.68) | 0.28 (0.41) | 2.20 (0.66) | 1.23 (0.86) | 2.30 (0.70) | 1.70 (0.70) |

We defined 6 areas of interest, which could clearly be identified in every sample and by every investigator (see additional Figure 2) :

- stratum basalaris as basal cells of the epidermis,

- stratum spinosum as row of cells close to the stratum basilaris,

- the seborrhoic glands,

- cells surrounding the hair, named as hairholder,

- cell infiltrate in the dermis below the scar,

- and, eventually the thickened layer of the epidermal regeneration as top of the scar.
