# Supplementary material for: Evaluation of the collaborative network of highly correlating skin proteins and its change following treatment with glucocorticoids
Source: Theor Biol Med Model. 2010 May 28;7:16. doi: 10.1186/1742-4682-7-16 (PMC2901312; doi:10.1186/1742-4682-7-16)
Supplement: Additional file 5 — Table S2: Calculation of Spearman's correlation coefficients revealed significant relations of several markers with therapy (positive r means increasing expression for hd-gc > ld-gc > ethanol, negative r means decreased expression for hd-gc > ld-gc > ethanol); * not confirmed by multivariate analysis, ## in case of significant impact in multivariate analysis, only. [file 1742-4682-7-16-S5.DOC]

**Additional Table 2:** Calculation of Spearman's correlation coefficients revealed significant relations of several markers with therapy (positive r means increasing expression for hd-gc>ld-gc>ethanol, negative r means decreased expression for hd-gc>ld-gc>ethanol), * if not confirmed by multivariate analysis, ## if only significant impact in multivariate analysis.

|  | **S basale** | **S spinosum** | **Seborrhoic gland** | **hairholder** | **Infiltrate** | **thickening** |
| --- | --- | --- | --- | --- | --- | --- |
| AXL |  |  | -0.485 | ## |  |  |
| Catenin | 0.791 | 0.632 |  |  |  |  |
| CD 68 |  |  |  |  | -0.73 |  |
| c-myc |  |  | -0.385* | -0.523 |  | -0.474 |
| Cox | 0.514 | 0.408 | -0.662 |  |  |  |
| ESDN |  | -0.669 | -0.408 |  | -0.408 | -0.729 |
| GAS6 |  |  | -0.608 |  |  |  |
| Ki67 |  | 0.529 | -0.610 | -0.717 |  |  |
| MMP-2 | 0.793 | 0.676 |  |  | 0.589 |  |
| Notch | 0.582 | 0.487* |  |  |  |  |
| S100 | 0.561* | -0.499 | -0.445 | -0.583 | -0.401 | -0.363 |
| SMA |  | -0.729 | ## | -0.479 | -0.702 | -0.669 |
| TGF |  |  | -0.781 | ## | 0.407 | -0.428 |
| TNF-R2 |  |  | -0.809 |  | 0.566 |  |
| TUNEL |  |  | -0.548 |  | -0.723 |  |
| Total number | 5 | 8 | 10 | 4 | 8 | 5 |
